# Dual-Domain Shape–Polarization Optical Encoding for Detector-Multiplexed Wide-Field Infrared Small-Target Imaging

**DOI:** 10.3390/s26185790

**Published:** 2026-09-12

**Authors:** Siqi Zhao, Zibo Yu, Guanyu Mu, Zhenyuan Guo, Bing Cao, Fei Liu, Yao Meng

**Affiliations:** 1 School of Optoelectronic Engineering, Changchun University of Science and Technology, Changchun 130013, China; 2025100412@mails.cust.edu.cn (S.Z.); 2024100479@mails.cust.edu.cn (B.C.); 2025100414@mails.cust.edu.cn (F.L.); 2 Changchun Institute of Optics, Fine Mechanics and Physics, Chinese Academy of Sciences, Changchun 130033, China; yuzibo22@mails.ucas.ac.cn (Z.Y.); muguanyu23@mails.ucas.ac.cn (G.M.); guozhenyuan23@mails.ucas.ac.cn (Z.G.); 3 University of Chinese Academy of Sciences, Beijing 101408, China

**Keywords:** detector-multiplexed imaging, infrared small-target imaging, point spread function engineering, polarization coding, computational optical imaging

## Abstract

Detector-multiplexed wide-field infrared imaging reuses detector area by folding several sub-fields of view onto a common focal-plane region, but the branch identity of an aliased small target is then unavailable from intensity alone. We propose a dual-domain optical code that combines a fixed Zemax-derived point-spread-function (PSF) library with a conditionally assigned linear-polarization codebook. To replace the earlier feature-level validation, we developed an image-level radiometric simulator that generates four analyzer images under a fixed incident-photon budget, applies mirror transfer, throughput loss, Poisson shot noise, background, read noise, and physical multi-target PSF superposition, and then re-extracts morphology and Stokes observables. The 1037 PSF samples were divided by sub-field into construction, calibration, and locked test sets of 623, 207, and 207 samples. The polarization-to-field assignment was selected by an exhaustive 9! search on the construction data, whereas the compound weight and separability threshold were fixed from calibration data only. In 25,000 locked target-level tests, the equal-weight-form compound decoder achieved 95.936% accuracy (95% confidence interval of 95.684–96.174%), compared with 10.836%, 45.564%, and 88.720% for intensity-only, shape-only, and polarization-only decoding. At the calibrated threshold, the effective capacities were five, four, and nine for the shape, polarization, and compound code spaces. The results are simulation-phase evidence and require experimental verification with measured infrared throughput and component tolerances.

## 1. Introduction

Wide-field infrared optical imaging systems are often required to observe a large angular range while retaining sufficient sampling, sensitivity, and localization accuracy for weak small targets. Simply increasing the focal-plane format is not always attractive because large cooled infrared arrays increase cost, cooling demand, readout bandwidth, and instrument volume [1,2]. These tradeoffs are also consistent with classical electro-optical imaging-system analysis, where detector sampling, optical throughput, noise, and field coverage must be balanced rather than optimized independently [3]. Optical detector multiplexing offers an alternative route: several sub-fields of view (sub-FOVs) can be folded onto a common detector region so that the detector area is reused. The central optical question is then not only how to form an image, but how to preserve the sub-FOV identity of each target after intentional aliasing.

From an information-encoding perspective, detector multiplexing is useful only if the folded optical paths carry distinguishable and calibratable physical labels after superposition. This viewpoint is aligned with computational optical imaging, in which optical encoding and digital reconstruction are designed as a coupled information-processing chain rather than as two independent stages [4,5,6,7]. Pure intensity measurements provide limited labeling capacity because multiple sub-FOVs share the same detector coordinates, and small-target measurements are easily affected by low photon flux, background structure, detector sampling, and optical tolerances. A practical multiplexed infrared imager therefore needs physical labels that are compact in hardware, stable under calibration, compatible with target-level detection, and recoverable from noisy analyzer measurements.

Two physically interpretable encoding dimensions motivate the present work. In our previous field-of-view shape-modulation study, a cylindrical modulation mechanism was used to assign field-dependent PSF morphology to different sub-FOVs, allowing deformed small-target recognition and spatial inversion in aliased images [8]. This approach treats controlled aberration as an information-bearing optical label, in line with wavefront coding and PSF engineering concepts [9,10,11]. In parallel, polarization-resolved imaging has shown that vector-domain measurements can provide information beyond scalar intensity imagery [12,13,14,15,16]. In our previous polarization-encoding study, Stokes-vector codes were used to separate detector-multiplexed infrared targets [17]. These two directions suggest that aberration-modulated morphology and polarization state can be co-designed as complementary optical code domains.

Each single-domain code has a natural failure mode in a folded infrared imaging chain. Shape coding can weaken under PSF similarity, low signal-to-noise ratio (SNR), detector sampling, and long multi-target overlap, whereas polarization coding is sensitive to analyzer noise, modulation error, partial polarization, and mirror-induced degradation in folded infrared paths [18,19,20,21]. These error sources are not identical: a condition that makes the PSF descriptor ambiguous does not necessarily destroy the Stokes label, and a polarization-transfer mismatch does not necessarily erase the field-dependent PSF morphology. The proposed method therefore assigns each sub-FOV a compound code consisting of a PSF-shape label and a Stokes-vector label.

**Figure 1:**
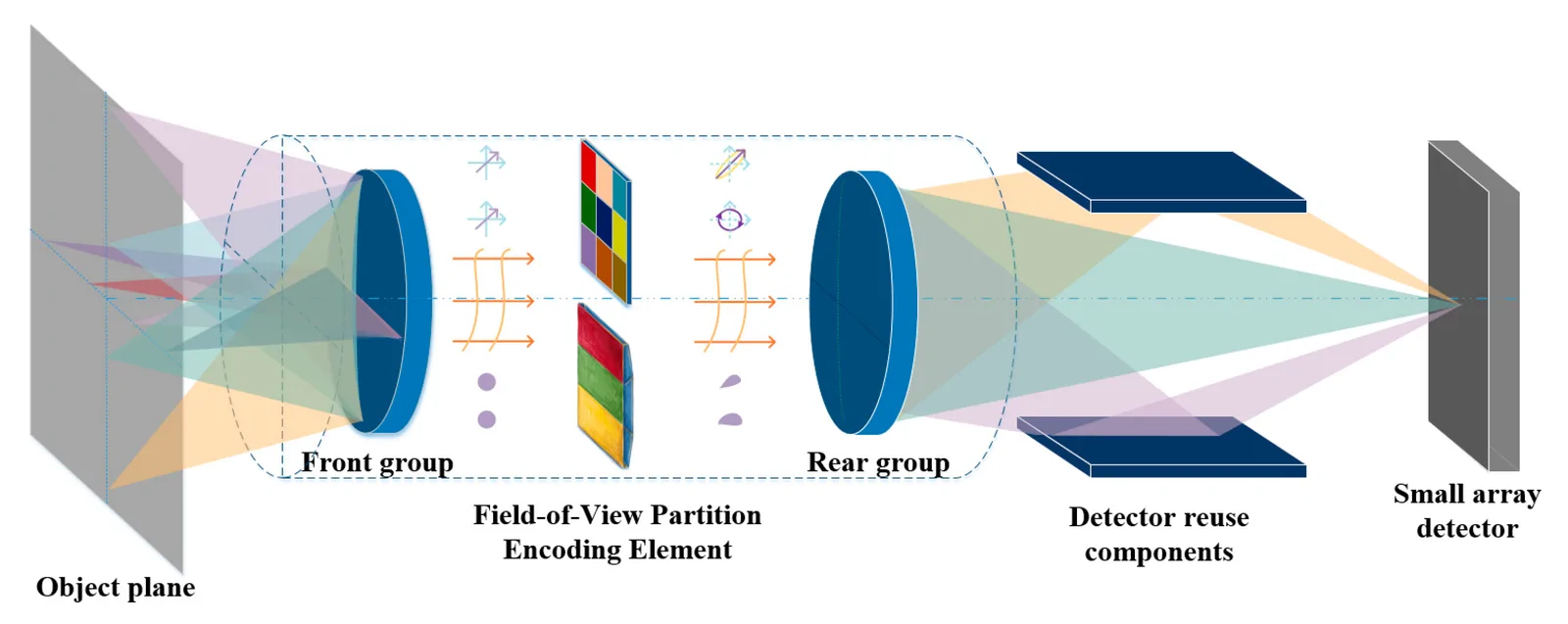
System-level optical schematic of the proposed dual-domain detector-multiplexed infrared imaging architecture. Nine sub-FOV regions are optically folded onto a shared detector region while two complementary physical labels are embedded before superposition. A cylindrical or aberration-modulation element produces field-dependent PSF morphology for shape-domain coding, whereas a polarization coding layer assigns Stokes-vector labels that are transported through the folded optical path by channel-dependent Mueller matrices. The analyzer channels reconstruct detector-side polarization states, and the shared detector image preserves both PSF-shape and Stokes-domain information for FOV discrimination.

The contribution of this work is a calibrated compound-code framework rather than a claim that the cylindrical PSF itself was jointly re-optimized with the polarization layer. First, the fixed, physically feasible Zemax PSF library is paired with nine distinct linear Stokes states, and their assignment to the nine folded branches is searched exhaustively. Second, a single normalized compound distance is used for code design, decoding, and capacity analysis. Third, the complete validation chain operates on noisy analyzer images and uses independent construction, calibration, and locked test partitions. This design tests whether complementary physical labels improve branch recovery under a fixed photon and detector-resource budget. The overall system-level architecture is illustrated in Figure 1.

## 2. Dual-Domain Optical Model for Detector-Multiplexed Infrared Imaging

**Figure 2:**
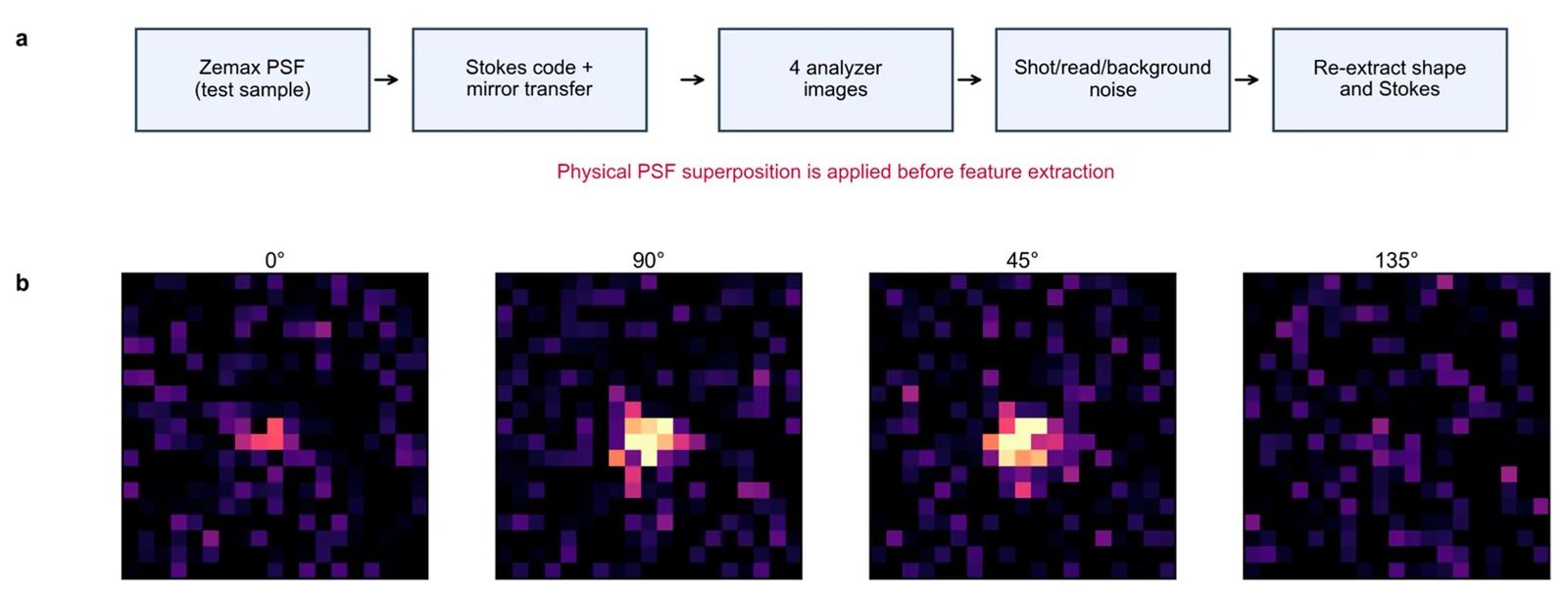
Image-level numerical verification chain. (**a**) Processing flow in which a locked Zemax PSF sample and a field-dependent linear Stokes code are propagated through the mirror model and projected into 0°, 90°, 45°, and 135° analyzer images; throughput, shot noise, background, and read noise are applied before morphology and Stokes features are re-extracted. (**b**) Representative analyzer-channel images after radiometric and noise modeling; the colors indicate relative image intensity only. When a second target is present, its analyzer-channel PSF is added on the image plane before feature extraction.

This section establishes the dual-domain optical forward model used throughout the paper. The model first describes how multiple sub-FOVs are folded onto a shared detector region, then introduces two complementary physical labels: a field-dependent PSF-shape descriptor and a mirror-aware Stokes-vector descriptor. These components are finally combined into a unified detector measurement equation, which provides the physical basis for the joint decoder developed in Section 3. The corresponding image-level numerical verification chain is summarized in Figure 2.

### 2.1. Detector Multiplexing with Compound Optical Labels

Consider a wide FOV divided into K sub-FOV regions. In a conventional staring system, each region would be mapped to a distinct detector area. In a detector-multiplexed system, the optical folding module maps these K regions onto a shared detector region. Let k denote the sub-FOV index. The target radiance distribution in the kth sub-FOV is denoted by $O_{k}(u,v,t)$, where (u, v) are object-space coordinates and t is the frame index.

In the proposed architecture, each sub-FOV is assigned a compound physical code

(1) $$C_{k}=\left\{C_{k}^{s},C_{k}^{p}\right\}$$

where the first term is the shape-domain code determined by the field-dependent PSF, and the second term is the polarization-domain code determined by the assigned Stokes vector. The purpose of the compound optical code is to preserve the FOV identity of each target after optical folding and detector superposition.

The shape-domain code can be represented by a feature vector

(2) $$C_{k}^{s}=[\phi_{k},\rho_{k},a_{k},b_{k},\Delta x_{k},\Delta y_{k}{]}^{T}$$

where $\phi_{k}$ is the principal-axis angle of the PSF, $\rho_{k}$ is the aspect ratio, $a_{k}$ and $b_{k}$ are effective semi-axis parameters, and $\Delta x_{k}$ and $\Delta y_{k}$ denote field-dependent centroid shifts. These quantities can be obtained from optical simulation, calibration measurements, or a hybrid optical–digital PSF library.

The polarization-domain code is represented by a normalized Stokes vector

(3) $$C_{k}^{p}=[1,q_{k},u_{k},v_{k}{]}^{T}$$

where $q_{k}=S_{1}/S_{0}$, $u_{k}=S_{2}/S_{0}$, and $v_{k}=S_{3}/S_{0}$. For a fully polarized state, the normalized Stokes vector can also be parameterized by the polarization azimuth $\psi_{k}$ and ellipticity angle $\chi_{k}$:

(4) $$q_{k}=\cos\left(2\psi_{k}\right)\cos\left(2\chi_{k}\right),u_{k}=\sin\left(2\psi_{k}\right)\cos\left(2\chi_{k}\right),v_{k}=\sin\left(2\chi_{k}\right)$$

The compound code therefore embeds the sub-FOV index into two complementary physical spaces: focal-plane morphology and polarization state.

### 2.2. Spatially Variant PSF-Shape Formation

A cylindrical modulation element placed near an intermediate image plane introduces field-dependent astigmatism, coma, and field curvature. Under this modulation, the PSF is no longer shift-invariant. Such field-dependent aberration behavior can be analyzed using classical aberration theory and nodal aberration concepts [22,23]. For the kth sub-FOV, the local image formation model can be approximated as.

(5) $$I_{k}^{s}\left(x,y,t\right)=h_{k}\left(x,y;\theta_{k}\right)*O_{k}\left(x,y,t\right)+n_{k}\left(x,y,t\right)$$

where $h_{k}$ is the local PSF, $\theta_{k}$ denotes the field-dependent aberration parameters, and * denotes local convolution under an isoplanatic approximation. For a small infrared target, $O_{k}$ can often be approximated as a point or compact Gaussian-like source. The observed target spot is therefore dominated by $h_{k}$.

The second-order central moments of the spot are

(6) $$\mu_{pq}=\frac{\sum_{x,y}(x-\bar{x}{)}^{p}(y-\bar{y}{)}^{q}I(x,y)}{\sum_{x,y}I(x,y)}$$

The principal-axis angle and effective semi-axes are obtained as

(7) $$\phi =\frac{1}{2}\mathrm{atan}2\left(2\mu_{11},\mu_{20}-\mu_{02}\right)$$

(8) $$a,b=\sqrt{\frac{\mu_{20}+\mu_{02}}{2}\pm \sqrt{{\left(\frac{\mu_{20}-\mu_{02}}{2}\right)}^{2}+\mu_{11}^{2}}}$$

These shape features form a compact geometric descriptor for FOV discrimination. Because the modulation is generated by deterministic field-dependent aberrations, the descriptor can be calibrated as a function of field angle. The use of locally calibrated PSFs is also consistent with spatially variant image restoration theory [24].

### 2.3. Polarization Encoding and Analyzer-Channel Measurement

In the polarization branch, each sub-FOV is assigned a Stokes code. After optical folding and polarization transfer, the detector receives a transported Stokes vector. This formulation follows standard Stokes–Mueller polarimetry:

(9) $$S_{k}^{d}=M_{k}^{opt}S_{k}^{e}$$

where $S_{k}^{e}$ is the encoder-side Stokes vector, $S_{k}^{d}$ is the detector-side Stokes vector, and $M_{k}^{opt}$ is the Mueller matrix of the optical path for the kth folded channel. In an ideal system $M_{k}^{opt}$ is the identity matrix. In a practical folded infrared system, $M_{k}^{opt}$ includes mirror-induced diattenuation and retardance, and therefore cannot be ignored.

For an analyzer channel m with Mueller analysis vector $a_{m}$, the measured intensity is

(10) $$Y_{m}\left(x,y,t\right)=\frac{1}{2}a_{m}^{T}S\left(x,y,t\right)+n_{m}\left(x,y,t\right)$$

The primary resource-aware configuration uses four simultaneous linear-analyzer channels at 0 deg, 90 deg, 45 deg, and 135 deg. These measurements recover $S_{0}$, $S_{1}$, and $S_{2}$, which are sufficient for the nine linear encoder states used here. A six-channel full-Stokes alternative that additionally measures right- and left-circular components is evaluated under the same incident-photon budget as a detector-resource comparison, not as a cost-free upper bound. The Stokes vector is reconstructed from the calibrated instrument matrix by linear least squares.

(11) $$\hat{S}(x,y,t)=(A^{T}A{)}^{-1}A^{T}Y(x,y,t)$$

Here, A is the measured instrument matrix and Y contains the background-subtracted analyzer-channel energies. Practical deployment requires calibration after the folding optics because mirror diattenuation and retardance are part of the measurement operator [25,26]. The polarization mask imposes the field label; no stable intrinsic target polarization is assumed. Throughput and analyzer-resource costs are explicitly included in Section 5.3.

### 2.4. Unified Forward Model

The complete detector-multiplexed measurement combines spatial folding, PSF-shape modulation, polarization transfer, and analyzer-channel projection. The mth analyzer-channel image can be written as:

(12) $$\;Y_{m}\left(x,y,t\right)=\sum_{k=1}^{K}P_{m,k}\left(M_{k}^{\mathrm{opt}}S_{k}^{e}\right)\left[h_{k}\left(x,y;\theta_{k}\right)*O_{k}\left(x,y,t\right)\right]+n_{m}\left(x,y,t\right)$$

where $P_{m,k}(\cdot )$ denotes the intensity projection of the transported Stokes vector onto analyzer channel m. This equation shows that the detector measurement is not merely a superposition of intensities. Each sub-FOV contribution carries both a PSF-shape signature and a polarization-state signature.

For each detected small target, the local observation is summarized by the re-extracted morphology vector and the reconstructed linear Stokes vector. Trajectory information is excluded from the primary comparison so that every method uses the same single-target evidence and detector resources.

(13) $$z_{t}={\left[f_{s}(t),\hat{S}(t)\right]}^{T}$$

The decoding task is to infer the sub-FOV origin from these two observables. Each Monte Carlo sample represents one target-level trial; no pixel-level accuracy or unimplemented temporal cue is reported.

## 3. Compound-Code Assignment and Calibrated Decoding

This section separates the fixed optical evidence from the quantities that are selected computationally. The Zemax PSF samples and the nine feasible linear-polarization states are fixed first. Only the assignment of polarization states to the nine PSF regions, the normalized fusion weight, and the separability threshold are selected. Construction data are used for code construction, calibration data are used for hyperparameters, and the test set remains locked.

### 3.1. Shape-Domain Code Separability

A cylindrical optical element possesses optical power only along one axis. If the cylindrical focal length is $f_{c}$, its optical power is:

(14) $$\Phi_{c}\;=\;\frac{1}{f_{c}},\;\;\;\Phi_{⊥}\;=\;0$$

Here, the first term denotes the optical power along the powered meridian of the cylindrical element, whereas the orthogonal meridian has no focusing power. This anisotropic optical power is the physical origin of the field-dependent astigmatic PSF morphology used by the shape-domain code.

The shape-domain distance between an observed spot descriptor $f_{s}$ and the calibrated descriptor $C_{k}^{s}$ is defined as

(15) $$d_{s}(k)=(f_{s}-C_{k}^{s}{)}^{T}\Sigma_{s,k}^{-1}(f_{s}-C_{k}^{s})$$

where $\Sigma_{s,k}$ is the covariance matrix of shape-feature uncertainty for the kth sub-FOV. The covariance accounts for photon noise, detector sampling, background clutter, PSF-library error, and centroid-estimation uncertainty.

Two shape codes $C_{i}^{s}$ and $C_{j}^{s}$ are highly separable if their Mahalanobis distance is large:

(16) $$D_{s}(i,j)=(C_{i}^{s}-C_{j}^{s}{)}^{T}(\Sigma_{s,i}+\Sigma_{s,j}{)}^{-1}(C_{i}^{s}-C_{j}^{s})$$

In PSF-shape encoding, the code design is constrained by optical feasibility. Excessive aberration can improve code diversity but may reduce energy concentration and target detectability. Therefore, shape-domain coding requires a balance between distinguishability and signal concentration.

### 3.2. Polarization-Domain Code Separability

The polarization-domain similarity between the reconstructed normalized Stokes vector $\hat{s}$ and the kth reference code $c_{k}^{p}$ is defined as:

(17) $$\gamma_{p}(k)=\frac{{\hat{s}}^{T}c_{k}^{p}}{∥\hat{s}∥∥c_{k}^{p}∥}$$

Equivalently, the polarization distance can be defined as:

(18) $$d_{p}\left(k\right)=1-\gamma_{p}\left(k\right)$$

For mirror-aware decoding, the detector-side reference should be

(19) $$c_{k,d}^{p}=\mathrm{normalize}\left(M_{k}^{opt}c_{k,e}^{p}\right)$$

Rather than the ideal encoder-side Stokes vector. If pre-compensation is used, the encoder-side vector is designed as:

(20) $$c_{k,e}^{p}=\mathrm{normalize}((M_{k}^{opt}{)}^{-1}c_{k,target}^{p})$$

Provided that the recovered state is physically valid. This allows the detector-side output to match the desired codebook even after mirror reflection. The same calibration principle is widely used in Mueller-matrix polarimetry, where the optical train must be treated as part of the measurement operator [27].

The polarization codebook should maximize pairwise angular distances on the Poincare sphere while avoiding states that become too close after optical propagation. A practical objective is

(21) $$\underset{\left\{c_{k}^{p}\right\}}{\max}\underset{i\neq j}{\min}\left[1-\frac{\left(M_{i}^{opt}c_{i}^{p}{)}^{T}(M_{j}^{opt}c_{j}^{p}\right)}{∥M_{i}^{opt}c_{i}^{p}∥∥M_{j}^{opt}c_{j}^{p}∥}\right]$$

This criterion makes the codebook robust to channel-dependent polarization transfer.

### 3.3. Conditioned Compound-Code Assignment

Let $D_{s,n}(i,j)$ and $D_{p,n}(i,j)$ denote the shape and polarization pairwise distances divided by their construction-set median pairwise distances. The same compound distance is used throughout this paper:

(22) $$D_{c}\left(i,j;\alpha \right)=\sqrt{\alpha D_{s,n}^{2}(i,j)+(1-\alpha )D_{p,n}^{2}(i,j)}$$

The fixed PSF code is not optically re-optimized in this study. For each candidate alpha, all 9! assignments of nine distinct linear states (polarization azimuths 0 deg to 160 deg in 20 deg increments) to the nine PSF regions are searched. The assignment that maximizes the minimum compound distance is retained. Alpha is then chosen only from the independent calibration trials.

(23) $$\pi^{*}\left(\alpha \right)=\underset{\pi \in \Pi }{arg\;max}\underset{i<j}{min}D_{c}\left(i,j;\alpha \right)$$

The search is therefore conditional on the measured PSF library, physically valid linear Stokes states, the calibrated mirror model, and the fixed analyzer architecture. It is an assignment optimization, not an end-to-end optimization of the cylindrical surface.

This conditional assignment places polarization states so that morphologically weak branch pairs receive stronger polarization separation. The exhaustive search and the final encoder- and detector-side codewords are reported in Table 1.

### 3.4. Normalized Compound-Distance Decoder

For a target observation, the shape residual to each branch is converted to a normalized Mahalanobis distance $d_{s,n}(k)$. The angular residual between the reconstructed and detector-side reference Stokes vectors gives $d_{p,n}(k)$. The primary decoder applies the same compound metric as the design stage and selects the minimum-distance branch:

(24) $$D_{k}=\sqrt{\alpha d_{s,n}^{2}(k)+(1-\alpha )d_{p,n}^{2}(k)},\hat{k}=\underset{k}{arg\;min}D_{k}$$

### 3.5. Calibration Protocol and Reliability Audit

Two observable confidence variables were audited without using test labels. Shape confidence combined the nearest-class distance margin, measured target SNR, and moment anisotropy. Polarization confidence combined the angular-distance margin, reconstructed degree of polarization, and analyzer-model residual. Monotonic logistic functions were fitted on calibration trials only.

(25) $$q_{s}=\Delta_{s}\sqrt{\mathrm{SNR}}\left[0.25+\frac{\lambda_{1}-\lambda_{2}}{\lambda_{1}+\lambda_{2}}\right];q_{p}=\frac{\Delta_{p}\mathrm{DoP}}{1+5\varepsilon_{A}}$$

After standardization, the fitted mappings were $\sigma (-0.304+0.489z_{s})$ for shape and $\sigma (2.890+1.512z_{p})$ for polarization. Their calibration-set Brier scores were 0.231 and 0.080, and their expected calibration errors were 0.010 and 0.003, respectively.

The calibrated reliability weights were frozen before testing. However, the adaptive variant reached 94.896% on the locked nominal test, below the 95.936% obtained by the simpler normalized compound distance. We therefore retain the equal-form compound decoder as the primary method and report the adaptive result only as a negative ablation.

This stop rule prevents a more complex fusion rule from being promoted without test evidence. All primary robustness and localization conclusions below therefore refer to the normalized compound-distance decoder of Section 3.4.

## 4. Numerical Verification Protocol

This section defines the reproducible image-level verification protocol. The pipeline uses the original Zemax PSF samples, explicit analyzer-channel photon formation, independent construction/calibration/test partitions, and identical target labels for all decoders.

### 4.1. Nine-Region Multiplexed FOV Configuration

A representative nine-region detector-multiplexed architecture was considered. The full FOV was divided into a 3 × 3 grid of sub-FOVs, and the optical folding module mapped all nine regions onto the same detector area. Each region was assigned a compound code consisting of one PSF-shape descriptor and one Stokes-vector descriptor.

**Figure 3:**
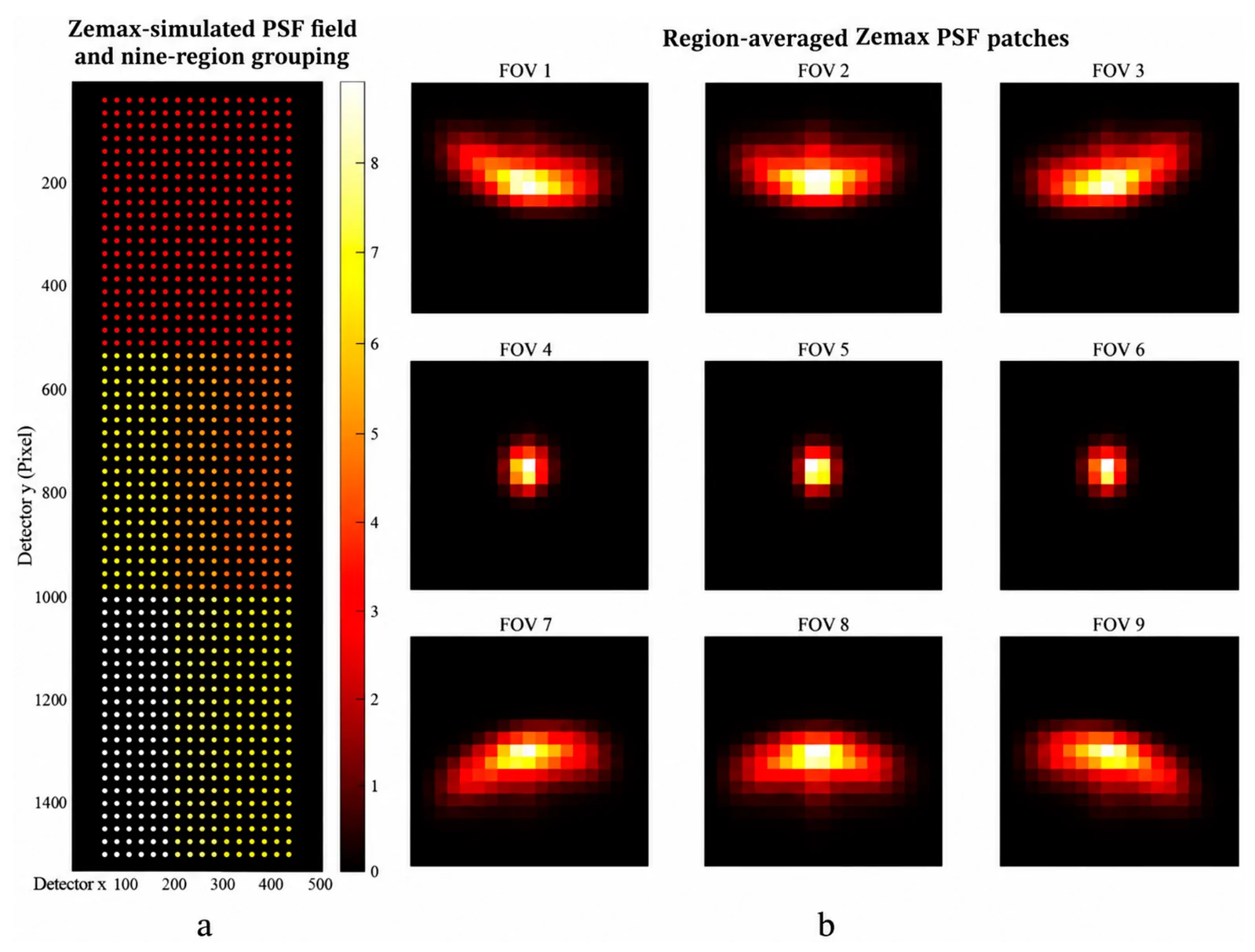
(**a**) Zemax-derived field sampling and nine-region PSF-code grouping. The simulated detector plane contains 1037 field samples generated from the wide-field optical model. Each sample is assigned to one of nine folded FOV regions, and local PSF patches are extracted around the theoretical image coordinates to construct the morphology codebook used in the dual-domain decoder. (**b**) Region-averaged Zemax PSF morphology used for shape-domain coding. Each panel shows the normalized mean PSF patch of one FOV region after local cropping and intensity normalization. The region-dependent orientation, elongation, effective area, and centroid bias form the calibrated shape-domain code.

The Zemax dataset contains 1037 field samples. The field sampling and nine-region grouping, together with the corresponding region-averaged PSF morphology, are shown in Figure 3. Within each of the nine spatial regions, samples were shuffled with seed 20,260,830 and split 60%/20%/20%, producing 623 construction, 207 calibration, and 207 locked test PSFs. Construction samples defined morphology centers, pooled covariance, normalization scales, and the conditioned code assignment. Calibration samples selected alpha and tau and fitted the reliability audit. Test PSFs were used only after all choices were frozen.

**Figure 4:**
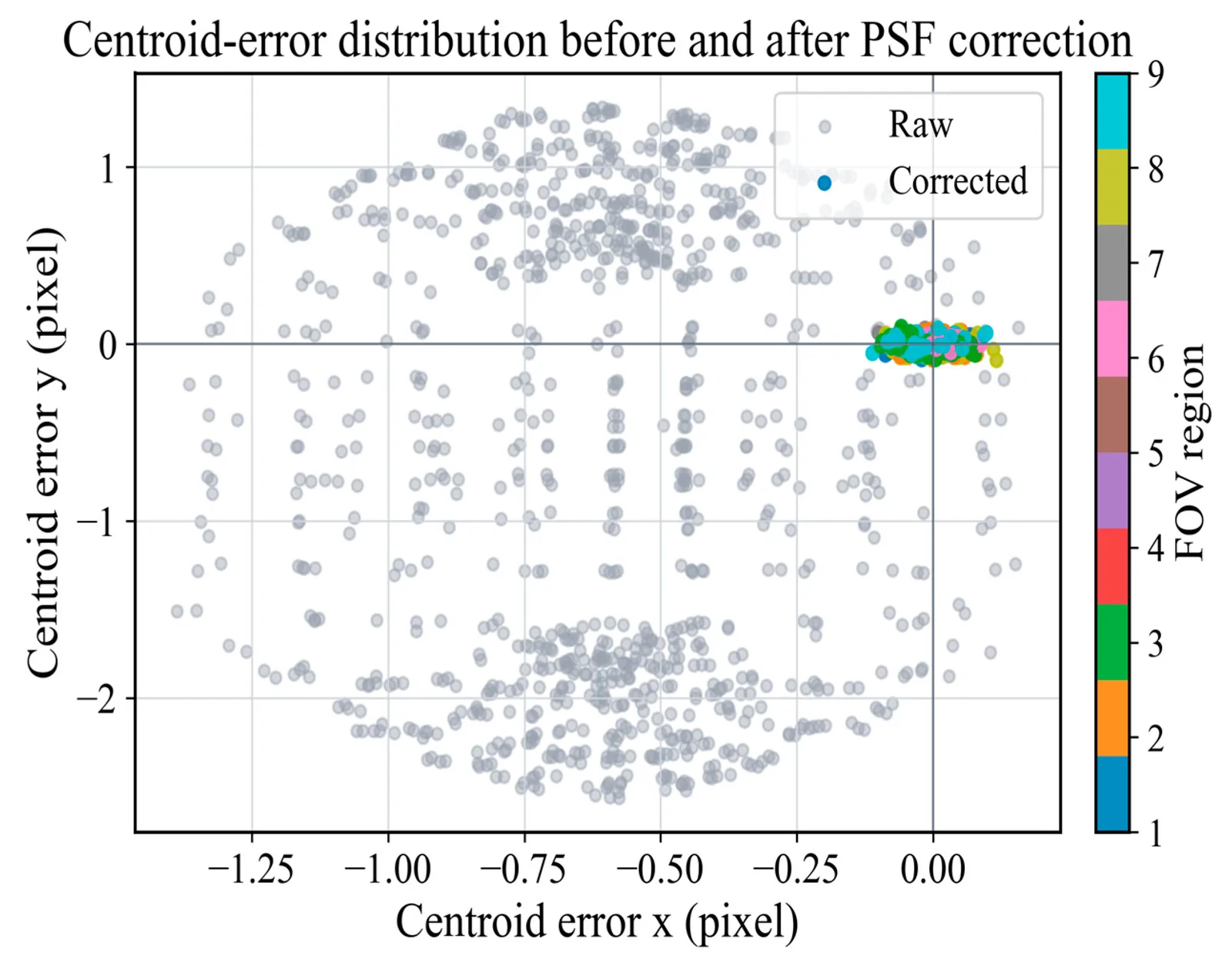
Centroid-error distribution before and after spatially variant PSF correction. Gray points denote raw centroid errors, and colored points denote deconvolution-corrected centroid errors grouped by FOV region. The tighter corrected distribution shows that the Zemax-derived PSF library provides a reliable continuous localization basis once the correct FOV branch is recovered.

**Figure 5:**
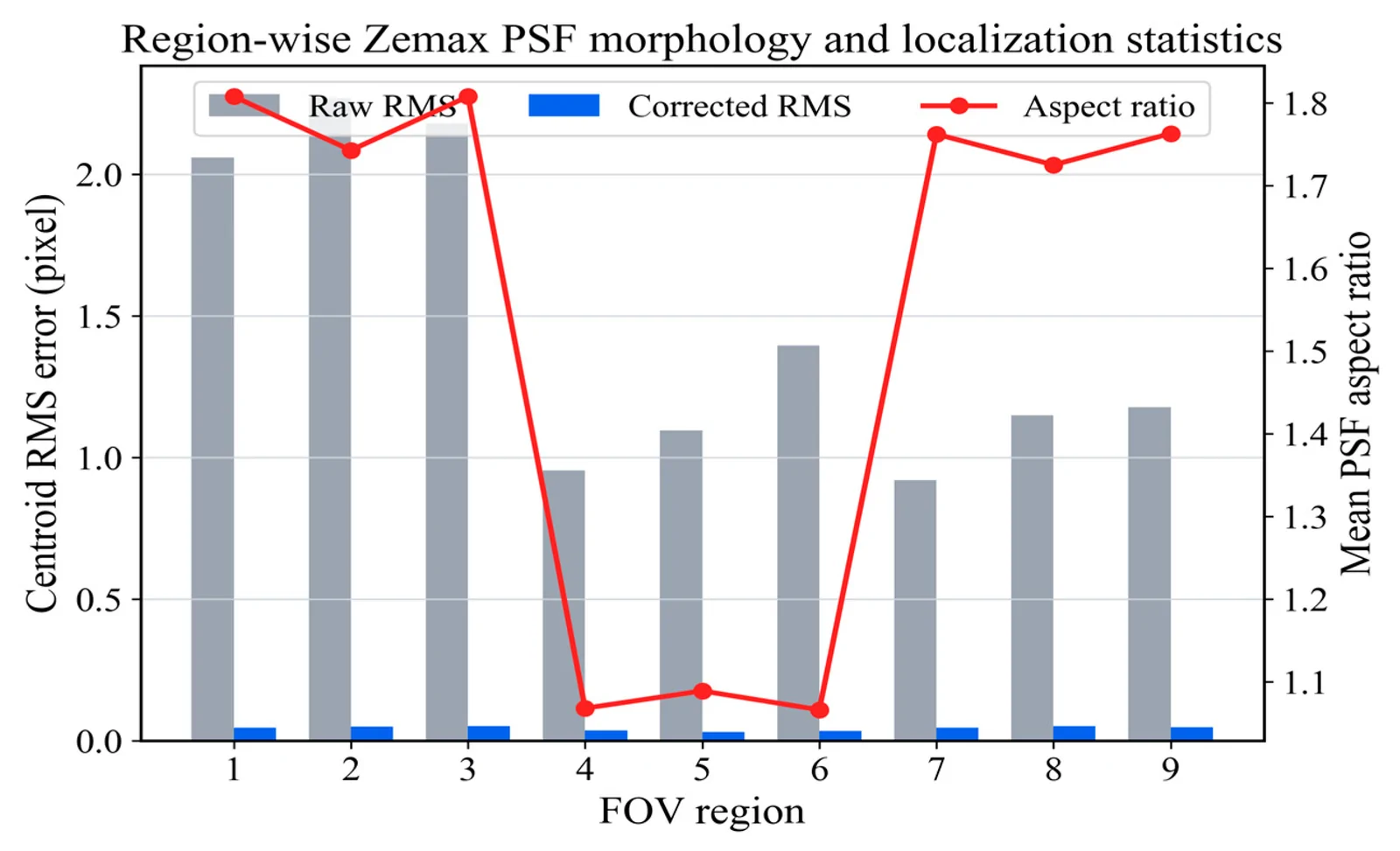
Region-wise Zemax PSF morphology and localization statistics. Bars show raw and deconvolution-corrected centroid RMS error for each FOV region, while the line curve gives the mean PSF aspect ratio. The result demonstrates that strong off-axis PSF deformation can be used as a discriminative physical code while still supporting subpixel localization after correction.

The primary encoder uses nine distinct linear states with azimuths separated by 20 deg. Their assignment to PSF regions was selected by exhaustive search for every candidate alpha. Detector-side references include the region-dependent mirror transfer at incidence angles of 58–70 deg. Table 1 reports the locked codebook; no codeword is duplicated in the Stokes space. The centroid-error distribution before and after spatially variant PSF correction is shown in Figure 4, while the region-wise PSF morphology and localization statistics are summarized in Figure 5.

### 4.2. Image-Level Radiometric and Perturbation Model

The nominal locked test contains 25,000 target-level trials with seed 20,260,901. Each trial draws a previously unseen test PSF and 1000 incident target photons. The four-channel architecture uses throughput eta = 0.46, background of 0.20 electron per pixel per channel, read noise 1.5 electrons rms, degree of polarization of 0.92, a 2 deg encoder perturbation, and a 0.75 deg residual mirror-reference rotation. Photon counts are sampled from a Poisson distribution before Gaussian read noise is added.

For overlap trials, a second target with 0.45–0.85 of the primary flux and a 1–3-pixel offset is propagated through its own PSF and analyzer coefficients. The two analyzer-image stacks are added before noise and before feature extraction. Thus, overlap is an image-level radiometric superposition rather than a mixture of precomputed descriptors. Single-factor sweeps use 8000 locked test trials per operating point with independent recorded seeds.

### 4.3. Benchmark Methods and Evaluation Metrics

Five audit rows are computed from identical target trials. The intensity-only method represents a detector-folding system with no branch label and therefore operates at the nine-class chance level. The shape-only and polarization-only methods use their respective normalized distances. The equal dual-domain method is the primary compound decoder. The calibrated adaptive variant is reported only to document the negative ablation described in Section 3.5. No method receives trajectory information.

FOV decoding is reported per target/trial, with Wilson 95% confidence intervals and a confusion matrix generated from the same 25,000 nominal predictions as Table 2. Localization uses the same predicted branch for every method. RMS, median, and 95th-percentile 2-D errors are computed in detector pixels.

Raw-oracle and deconvolution-oracle bounds use the correct branch and the original Zemax centroid residuals. For a wrong branch, the error includes the measured displacement between the corresponding regional median image coordinates. These oracles are reference floors, not competing branch decoders.

## 5. Numerical Results and Discussion

This section reports only results generated by the unified image-level pipeline. All tabulated accuracies, confusion matrices, robustness curves, capacity values, and localization metrics share the same partition definitions and locked configuration.

### 5.1. FOV Decoding Accuracy and Robustness

**Figure 6:**
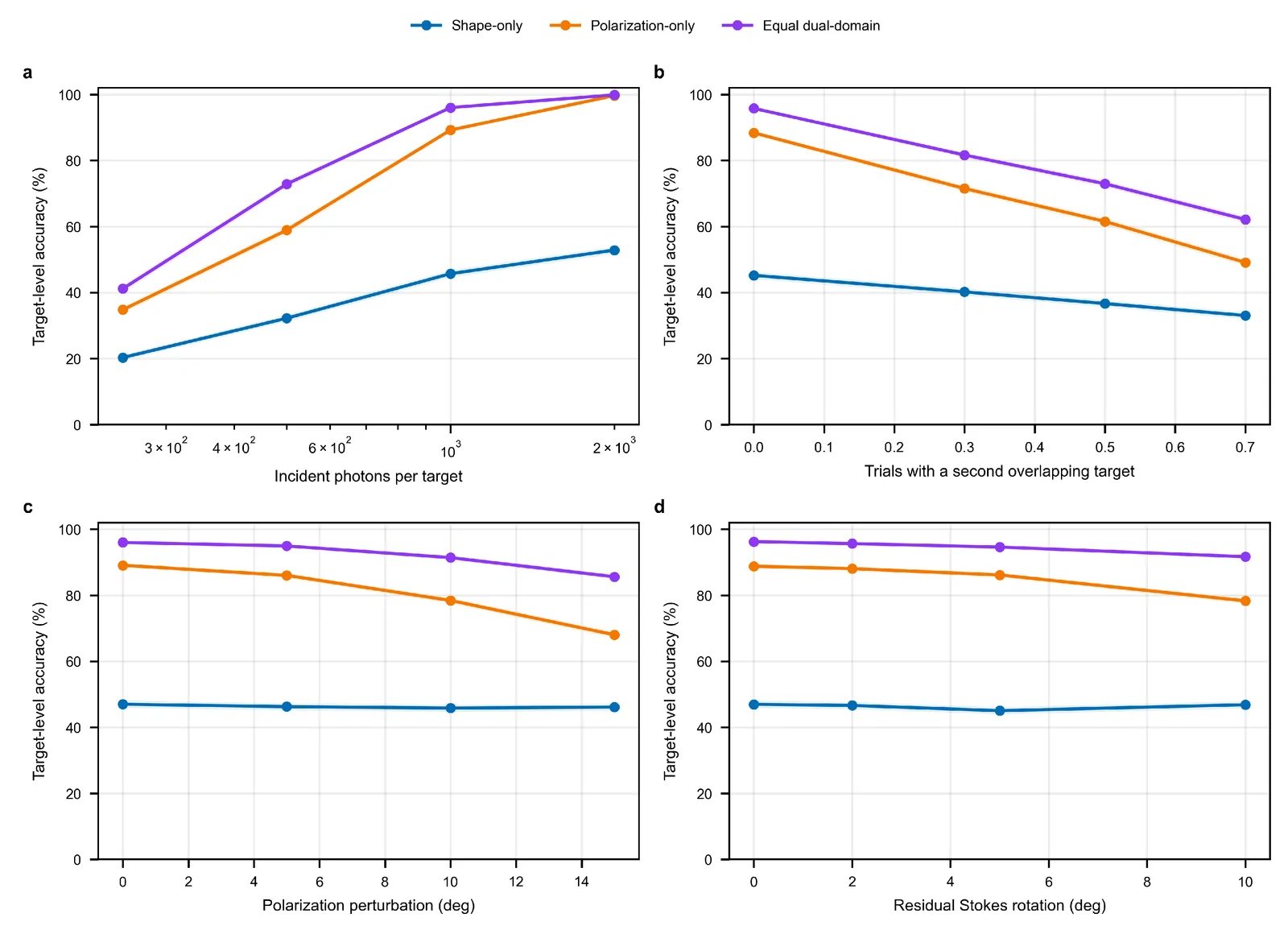
Locked-test robustness of target-level FOV classification. (**a**) Incident-photon budget under fixed four-channel throughput. (**b**) Probability that a second target is physically superposed in the analyzer images. (**c**) Additional encoder polarization perturbation. (**d**) Residual mirror-reference Stokes rotation. Each point contains 8000 independent target trials; shaded bands denote Wilson 95% confidence intervals.

On 25,000 locked nominal trials, intensity-only, shape-only, polarization-only, and equal dual-domain decoding achieved 10.836%, 45.564%, 88.720%, and 95.936%, respectively (Table 2). The compound result corresponds to 23,984 correct targets and a Wilson 95% confidence interval of 95.684–96.174%. Figure 6 shows that its advantage persists across photon budget, physical overlap, polarization perturbation, and mirror-reference mismatch.

At 250, 500, 1000, and 2000 incident photons, the compound accuracies were 41.175%, 72.863%, 96.025%, and 99.875%. The gain therefore depends on a sufficient photon budget and is not a claim of photon-free coding. At 50% and 70% physical-overlap probabilities, accuracy decreased to 72.950% and 62.138%, which defines a clear failure trend.

Additional polarization perturbations of 5 deg, 10 deg, and 15 deg produced compound accuracies of 94.938%, 91.400%, and 85.600%. Residual mirror-reference rotations of 2 deg, 5 deg, and 10 deg produced 95.650%, 94.600%, and 91.675%. The shape branch is insensitive to these polarization perturbations and therefore supplies complementary evidence.

The reliability audit did not improve the nominal result: adaptive fusion reached 94.896%, 1.040 percentage points below the equal-form compound decoder. This negative result is retained in Table 2 and is the reason adaptive weighting is not used for the primary claims.

**Figure 7:**
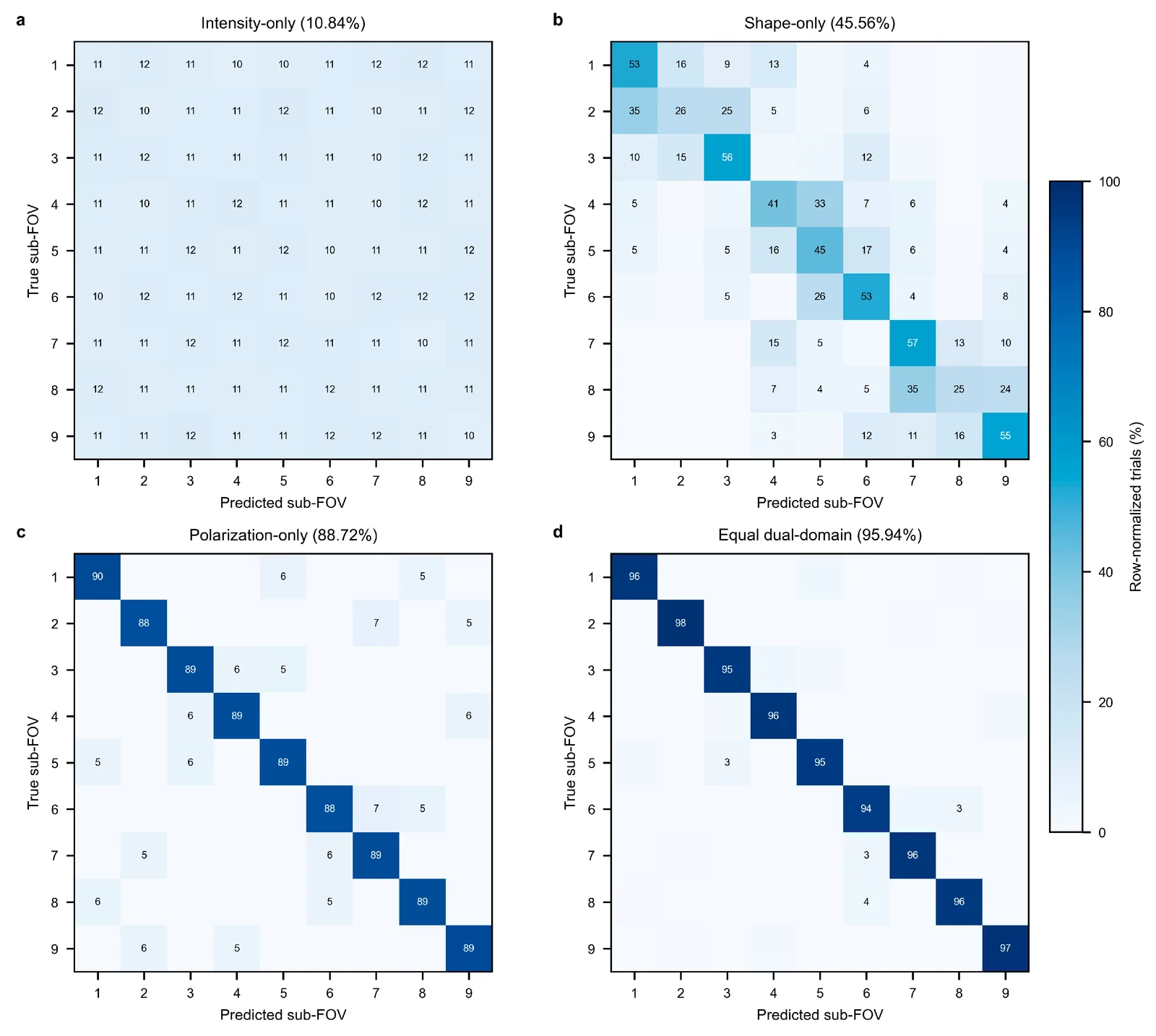
Row-normalized nine-region confusion matrices from the same 25,000 locked nominal target trials as Table 2. (**a**) Intensity only. (**b**) Shape only. (**c**) Polarization only. (**d**) Equal dual domain. Values below 3% are omitted for readability.

Figure 7 uses exactly the predictions summarized in Table 2. The intensity baseline is uniformly distributed, the shape matrix shows several adjacent-region ambiguities, and polarization-only decoding remains near 89% on most diagonal entries. The compound decoder concentrates every row between 94% and 98% on the correct branch.

### 5.2. Calibration, Code Capacity, and Localization

**Figure 8:**
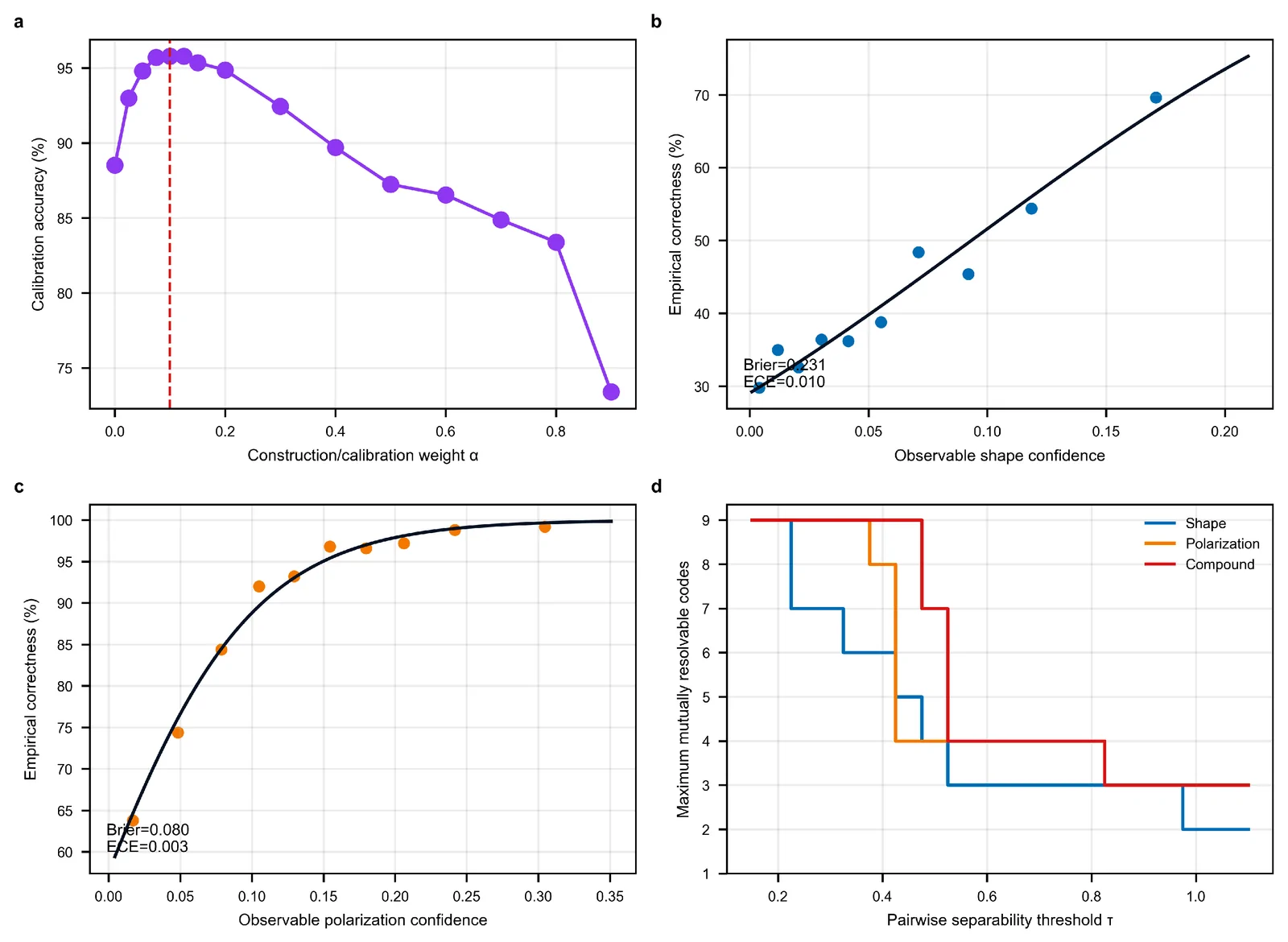
Calibration and capacity audit. (**a**) Calibration accuracy across the predeclared alpha grid; the red dashed line marks the selected α = 0.10. (**b**,**c**) Held-out monotonic reliability mappings with Brier score and expected calibration error. (**d**) Effective-capacity sensitivity to τ.

The calibration sweep selected alpha = 0.10; alpha = 0.125 gave the same rounded calibration accuracy, and the first maximum on the predeclared ascending grid was retained. A binomial logistic fit between pairwise compound distance and calibration binary accuracy set tau = 0.417 at a predicted 95% pairwise correctness level. Figure 8 reports the full alpha and tau sensitivity rather than only the selected values.

The raw- and deconvolution-oracle RMS errors were 1.543 and 0.043 detector pixels. Full branch-aware RMS errors were 248.554 pixels for the shape-only, 237.093 pixels for the polarization-only, and 119.620 pixels for the compound decoder. The compound median and 95th-percentile errors were 0.034 and 0.105 pixels because 95.936% of targets were assigned to the correct branch; the remaining branch failures dominate the RMS.

**Figure 9:**
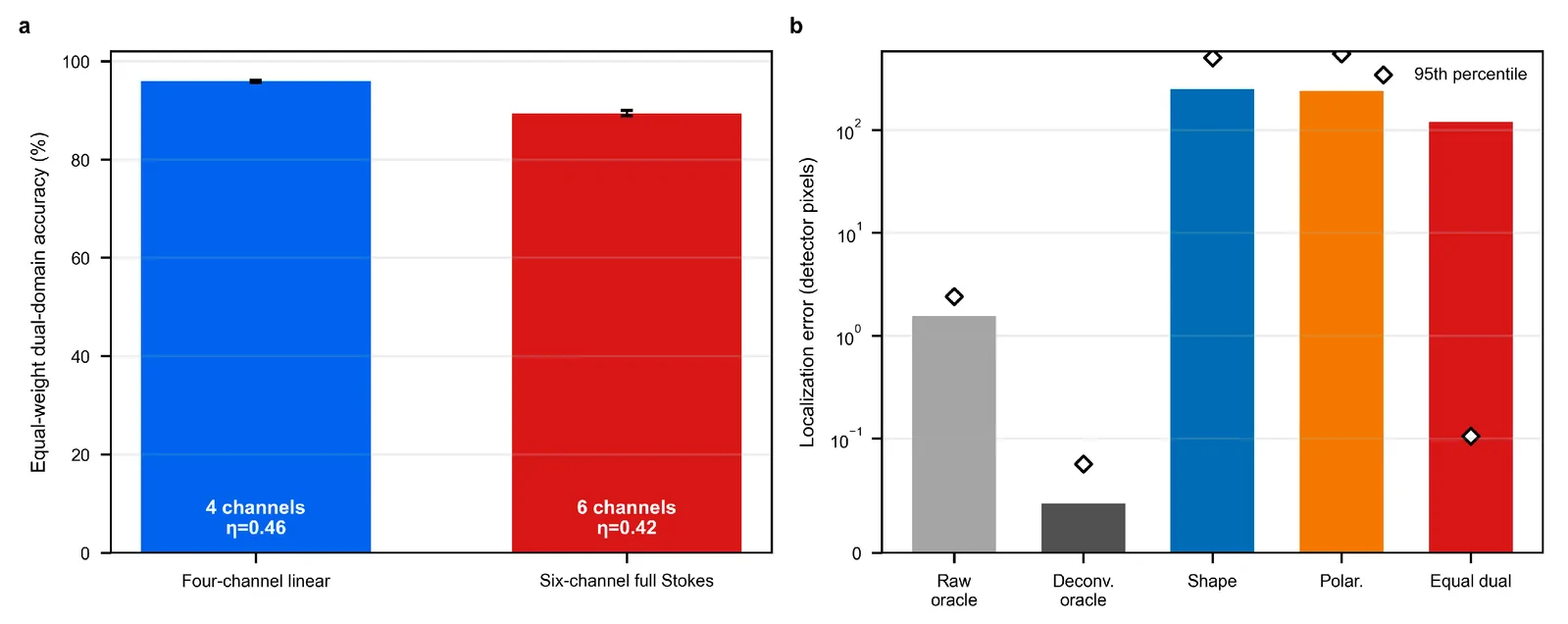
Detector-resource and localization audit. (**a**) Four-channel linear- and six-channel full-Stokes architectures under the same 1000-photon incident budget; error bars denote Wilson 95% confidence intervals. (**b**) Branch-aware RMS localization errors, with diamonds showing the 95th-percentile radial errors.

These metrics demonstrate why classification and localization must be reported together. A small fraction of branch errors produces a large RMS even when correctly assigned targets retain the deconvolution centroiding floor [28]. Figure 9b therefore shows both RMS bars and 95th-percentile markers.

Code capacity is evaluated with the same normalized compound distance defined in Equation (22). A code subset is resolvable at threshold tau only when every pair in the subset has distance at least tau.

At the calibration-derived tau = 0.417, the shape, polarization, and compound code spaces had seven, nine, and zero weak pairs. Their maximum mutually resolvable subsets contained five, four, and nine codes, respectively.

For the nine-region codebook, the weak-pair count and effective capacity are evaluated directly from Equation (22); no second definition of compound distance is introduced.

A field pair is weak when its normalized distance is below tau. The weak-pair count and maximum-clique effective capacity are:

(26) $$W_{x}\left(\tau \right)=\sum_{i<j}I\left[D_{x}(i,j)<\tau \right],x\in \left\{s,p,c\right\}$$

(27) $$C_{\mathrm{eff},x}\left(\tau \right)=\underset{V\subseteq \left\{1,\ldots ,K\right\}}{max}\left|V\right|\;\mathrm{subject}\;\mathrm{to}\;D_{x}\left(i,j\right)\geq \tau \;\mathrm{for}\;\mathrm{all}\;i<j\in V$$

The minimum normalized distances were 0.219, 0.390, and 0.485 for shape, polarization, and compound coding, respectively (Table 3). The compound distance lifted every pair above the calibrated threshold and recovered all nine branches as a mutually resolvable set.

The capacity sensitivity in Figure 8d shows that the nine-code conclusion is specific to the calibrated threshold and is not asserted for arbitrarily strict separation criteria. This bounded interpretation replaces the earlier fixed-threshold claim.

### 5.3. Practical Implementation Considerations

The primary architecture uses a cylindrical modulation element, nine linear encoder states, a folding module, and four simultaneous linear-analyzer channels. Under 1000 incident photons, the four-channel design with eta = 0.46 achieved 95.936%, whereas the six-channel full-Stokes alternative with eta = 0.42 achieved 89.433% in 12,000 locked trials. The additional circular channels did not offset their photon-splitting and readout cost in this configuration (Figure 9a).

This resource result motivates the reduced linear-Stokes implementation rather than a cost-free full-Stokes assumption. Actual throughput depends on coatings, fill factor, analyzer integration, and detector architecture [29]; the values used here are explicit simulation parameters, not universal hardware specifications.

The target need not possess a stable intrinsic polarization signature because the field code is imposed by the optical encoder. Polarized backgrounds, depolarization, stray light, and temperature drift can nevertheless alter the recovered Stokes vector and must be included in instrument-specific calibration [30,31].

The main limitation is that the present evidence remains simulation-phase. The PSFs are Zemax-derived, mirror transfer is modeled, and component throughput and detector noise are prescribed rather than measured in a complete infrared prototype. The current results therefore establish algorithmic and radiometric feasibility, not demonstrated hardware performance.

## 6. Conclusions

This paper presents a dual-domain shape–polarization code for detector-multiplexed wide-field infrared small-target imaging. The revised method conditions the polarization assignment on a fixed Zemax PSF library and uses a four-channel analyzer model with explicit photon, throughput, background, and read-noise accounting.

A single normalized compound distance now governs assignment, decoding, and capacity. Independent construction, calibration, and locked test partitions eliminate the earlier reuse of the same simulated population for design and reporting. The simpler equal-form decoder was retained because its 95.936% locked accuracy exceeded the calibrated adaptive ablation.

The compound code recovered all nine branches at the calibration-derived separability threshold and reduced nominal branch-induced localization RMS to 119.620 pixels. Performance decreased under low photon counts and frequent physical overlap, and the six-channel alternative was inferior under a fixed photon budget. These boundaries are part of the result. Future work will measure component throughput and Mueller matrices, fabricate the intermediate-image polarization encoder, and validate the complete pipeline on an infrared hardware prototype.

## Figures and Tables

**Table 1:** Locked four-channel linear-polarization codebook after conditioned assignment and mirror transfer.

Region	Encoder Azimuth (deg)	Encoder S1	Encoder S2	Detector S1	Detector S2
1	140.0	0.174	−0.985	0.162	0.987
2	60.0	−0.500	0.866	−0.511	−0.860
3	0.0	1.000	0.000	1.000	0.000
4	20.0	0.766	0.643	0.761	−0.649
5	160.0	0.766	−0.643	0.760	0.650
6	100.0	−0.940	−0.342	−0.943	0.334
7	80.0	−0.940	0.342	−0.943	−0.332
8	120.0	−0.500	−0.866	−0.514	0.858
9	40.0	0.174	0.985	0.161	−0.987

**Table 2:** Locked nominal target-level FOV-classification results.

Method	Correct/25,000	Accuracy (%)	95% CI (%)
Intensity-only	2709	10.836	10.457–11.227
Shape-only	11,391	45.564	44.947–46.182
Polarization-only	22,180	88.720	88.322–89.106
Equal dual-domain	23,984	95.936	95.684–96.174
Adaptive dual-domain	23,724	94.896	94.616–95.162

**Table 3:** Pairwise separability and effective capacity at the calibrated threshold tau = 0.417.

Code Space	Minimum Distance	Weak Pairs	Effective Capacity	Median Distance
Shape	0.219	7	5	1.000
Polarization	0.390	9	4	1.000
Compound	0.485	0	9	0.997

## Data Availability

The data presented in this study are available on request from the corresponding author.
